# Supplementary material for: Association between country preparedness indicators and quality clinical care for cardiovascular disease risk factors in 44 lower- and middle-income countries: A multicountry analysis of survey data
Source: PLoS Med. 2020 Nov 10;17(11):e1003268. doi: 10.1371/journal.pmed.1003268 (PMC7654799; doi:10.1371/journal.pmed.1003268)
Supplement: S1 Table — NCD, noncommunicable disease. (DOCX) [file pmed.1003268.s002.docx]

# **S1 Table. Summary of source of data for individual-level predictors and outcomes (e.g., cascades of care) relative to dates for higher-level predictors (e.g., NCD preparedness indicators)**

| Country | Survey used in cascade of care | Income status at time of the survey used in cascade of care | WHO NCD Report (year) | WHO NCD Report data collection (year) | SARA Report (year) | SARA Report data collection (year) | SPA Report (year) | SPA Report data collection (year) | WDI extraction date^1^ |
| --- | --- | --- | --- | --- | --- | --- | --- | --- | --- |
| Albania | DHS 2008 | Lower-middle | 2011 | 2010 |  |  |  |  | Sep-2018 |
| Azerbaijan | DHS 2006 | Lower-middle |  |  |  |  |  |  | Sep-2018 |
| Bangladesh | DHS 2011 | Low | 2011 | 2010 |  |  |  |  | Sep-2018 |
| Belize | CAMDI 2005/06 | Upper-middle | 2011 | 2010 |  |  |  |  | Sep-2018 |
| Benin | STEPS 2008 | Low | 2011 | 2010 |  |  |  |  | Sep-2018 |
| Bhutan | STEPS 2014 | Lower-middle | 2014 | 2013 |  |  |  |  | Sep-2018 |
| Brazil | PNS 2013 | Upper-middle | 2014 | 2013 |  |  |  |  | Sep-2018 |
| Burkina Faso | STEPS 2013 | Low | 2014 | 2013 | 2014 | 2014 |  |  | Sep-2018 |
| Chile | NHS 2009-10 | Upper-middle | 2011 | 2010 |  |  |  |  | Sep-2018 |
| China | CHNS 2009 | Lower-middle | 2011 | 2010 |  |  |  |  | Sep-2018 |
| Comoros | STEPS 2011 | Low | 2011 | 2010 |  |  |  |  | Sep-2018 |
| Costa Rica | STEPS 2010 | Upper-middle | 2011 | 2010 |  |  |  |  | Sep-2018 |
| Ecuador | ENSANUT 2012 | Upper-middle | 2014 | 2013 |  |  |  |  | Sep-2018 |
| Egypt | EHIS 2015 | Lower-middle | 2014 | 2013 |  |  |  |  | Sep-2018 |
| Fiji | EHS 2009 | Upper-middle | 2011 | 2010 |  |  |  |  | Sep-2018 |
| Georgia | STEPS 2016 | Lower-middle |  |  |  |  |  |  | Sep-2018 |
| Ghana | SAGE 2007-08 | Low | 2011 | 2010 |  |  |  |  | Sep-2018 |
| Grenada | STEPS 2010-11 | Upper-middle | 2011 | 2010 |  |  |  |  | Sep-2018 |
| Guyana | STEPS 2016 | Upper-middle |  |  |  |  |  |  | Sep-2018 |
| India | NFHS 2015-2016 | Lower-middle | 2014 | 2013 |  |  |  |  | Sep-2018 |
| Indonesia | IFLS 2014-15 | Lower-middle | 2014 | 2013 |  |  |  |  | Sep-2018 |
| Kazakhstan | HHS 2012 | Upper-middle | 2014 | 2013 |  |  |  |  | Sep-2018 |
| Kenya | STEPS 2015 | Lower-middle | 2014 | 2013 | 2013 | 2013 |  |  | Sep-2018 |
| Kyrgyzstan | DHS 2012 | Low | 2014 | 2013 |  |  |  |  | Sep-2018 |
| Lebanon | STEPS 2008 | Upper-middle | 2011 | 2010 |  |  |  |  | Sep-2018 |
| Lesotho | DHS 2014 | Lower-middle | 2014 | 2013 |  |  |  |  | Sep-2018 |
| Liberia | STEPS 2011 | Low | 2011 | 2010 |  |  |  |  | Sep-2018 |
| Mexico | MxFLS 2009-12 | Upper-middle | 2014 | 2013 |  |  |  |  | Sep-2018 |
| Mongolia | STEPS 2009 | Lower-middle | 2011 | 2010 |  |  |  |  | Sep-2018 |
| Mozambique | STEPS 2005 | Low |  |  |  |  |  |  | Sep-2018 |
| Namibia | DHS 2013 | Upper-middle | 2014 | 2013 |  |  |  |  | Sep-2018 |
| Nepal | STEPS 2013 | Low | 2014 | 2013 |  |  | 2015 | 2015 | Sep-2018 |
| Peru | DHS 2012 | Upper-middle | 2014 | 2013 |  |  |  |  | Sep-2018 |
| Romania | SEPHAR 2015-16 | Upper-middle | 2014 | 2013 |  |  |  |  | Sep-2018 |
| Russia | SAGE 2007-08 | Upper-middle | 2011 | 2010 |  |  |  |  | Sep-2018 |
| Saint Vincent and the Grenadines | STEPS 2013 | Upper-middle | 2014 | 2013 |  |  |  |  | Sep-2018 |
| Seychelles | STEPS 2013 | Upper-middle | 2014 | 2013 |  |  |  |  | Sep-2018 |
| South Africa | SANHANES 2012 | Upper-middle | 2014 | 2013 |  |  |  |  | Sep-2018 |
| Swaziland | STEPS 2014 | Lower-middle | 2014 | 2013 |  |  |  |  | Sep-2018 |
| Timor-Leste | STEPS 2014 | Lower-middle | 2014 | 2013 |  |  |  |  | Sep-2018 |
| Togo | STEPS 2010 | Low | 2011 | 2010 | 2012 | 2012 |  |  | Sep-2018 |
| Uganda | STEPS 2014 | Low | 2014 | 2013 | 2014 | 2013/2014 |  |  | Sep-2018 |
| Ukraine | DHS 2007 | Lower-middle |  |  |  |  |  |  | Sep-2018 |
| United Republic of Tanzania | STEPS 2012 | Low | 2014 | 2013 | 2013 | 2012 |  |  | Sep-2018 |

Abbreviations: CAMDI, Central America Diabetes Initiative; CHNS, China Health and Nutrition Survey; DHS, Demographic and Health Survey; EHS, Eye Health Survey; EHIS, Egypt Health Issues Survey; ENSANUT, La Encuesta Nacional de Salud y Nutrición; HHS, Household Survey Health; IFLS, Indonesia Family Life Survey; MxFLS, Mexico Family Life Survey; NCD, non-communicable disease; NFHS, National Family and Health Survey; NHS, National Health Survey; PNS, Pesquisa Nacional de Saúde; SAGE, Study on global AGEing and adult health; SARA, Service Availability Readiness Assessments; SANHANES, South Africa National Health And Nutrition Examination Survey; SEPHAR, Study for the Evaluation of Prevalence of Hypertension and Cardiovascular Risk; SPA, Service Provision Assessments; STEPS, WHO STEPwise approach to surveillance; WDI, World Development Indicators; WHO, World Health Organization.

^1^Data were extracted from the Wold Bank WDI data base in September 2018 for the year corresponding to the year of the country survey used in the cascade construction.
