# Supplementary material for: Association between country preparedness indicators and quality clinical care for cardiovascular disease risk factors in 44 lower- and middle-income countries: A multicountry analysis of survey data
Source: PLoS Med. 2020 Nov 10;17(11):e1003268. doi: 10.1371/journal.pmed.1003268 (PMC7654799; doi:10.1371/journal.pmed.1003268)
Supplement: S1 Text — (DOCX) [file pmed.1003268.s003.docx]

# **S1 Text: Information on data used in constructing the cascades**

# **Country specifc information on questionnaires used in constructing the cascade**

# Data to construct diabetes and hypertension cascades were extracted from STEPs or other similar surveys (see table S1). Methods and questionnaires used in each of these surveys can be found at the below sources:

# STEPs: <https://www.who.int/ncds/surveillance/steps/instrument/en/>

# DHS: <https://dhsprogram.com/What-We-Do/Survey-Types/SPA.cfm>

# SAGE: https://www.who.int/healthinfo/sage/en/Camdi at <https://iris.paho.org/handle/10665.2/7687>

# PNS, Brazil: [https: //www.ibge.gov.br/en/statistics/social/justice-and-security/16840-national-survey-of-health.html?edicao=19375&t=sobre](https://www.ibge.gov.br/en/statistics/social/justice-and-security/16840-national-survey-of-health.html?edicao=19375&t=sobre)

# NHS, Chile: <http://epi.minsal.cl/encuesta-ens-anteriores/>

# CHNS, China: https://www.cpc.unc.edu/projects/china

# ENSANU, Ecuador: <https://www.salud.gob.ec/encuesta-nacional-de-salud-y-nutricion-ensanut/>.

# EHIS, Egypt: https://dhsprogram.com/pubs/pdf/FR313/FR313.pdf

# EHS, Fij: https://www.tandfonline.com/doi/full/10.3109/09286586.2011.628135

# NFHS, India <http://rchiips.org/NFHS/factsheet_NFHS-4.shtml>

# IFLS, Indonesia: <https://www.rand.org/well-being/social-and-behavioral-policy/data/FLS/IFLS/download.html>

# SANHANES, South Africa: <http://www.hsrc.ac.za/en/research-areas/Research_Areas_PHHSI/sanhanes-health-and-nutrition>

# HHS, Khazakstan: <https://www.academypm.org/language/en/household-health-surveys/>.

# MxFLS, Mexico: http://www.ennvih-mxfls.org/english/ennhiv-3.html

# SEPHAR, Romania: http://www.mymed.ro/studiul-sephar.html

# The questions asked in each survey were similar to those asked in the STEPS survey, which the majority of countries employed. These are as below:

| Have you ever had your blood sugar/blood pressure measured by a doctor or other health worker? | Yes  No |
| --- | --- |
| Have you ever been told by a doctor or other health worker that you have raised blood sugar or diabetes/raised blood pressure or hypertension? | Yes  No |
| In the past two weeks, have you taken any drugs (medication) for diabetes/hypertension prescribed by a doctor or other health worker? | Yes  No |
| Are you currently taking insulin for diabetes prescribed by a doctor or other health worker? | Yes  No |
| During the past three years, has a doctor or other health worker advised you to do any of the following?  (RECORD FOR EACH) |  |
| Reduce fat in your diet | Yes  No |
| Start or do more physical activity | Yes  No |
| Maintain a healthy body weight or lose weight | Yes  No |

There was some variability across surveys in the specific questions asked including the recall period (e.g., “ever” versus “in the past 12 months” for tested).

# **Country-specific contact information regarding accessing cascade data used in this study**

Data included in this study are only publically available for 18 of the 44 countries. The links to where data can be downloaded (upon free registration) are:

Albania: https://dhsprogram.com/data/dataset/Albania_Standard-DHS_2008.cfm?flag=0

Azerbaijan: https://dhsprogram.com/data/dataset/Azerbaijan_Standard-DHS_2006.cfm?flag=1

Bangladesh: https://dhsprogram.com/data/dataset/Bangladesh_Standard-DHS_2011.cfm?flag=0

Brazil: https://www.ibge.gov.br/estatisticas/downloads-estatisticas.html

Chile: https://www.minsal.cl/estudios_encuestas_salud/

China: https://www.cpc.unc.edu/projects/china/data/datasets

Ecuador: https://ensanut.insp.mx/encuestas/ensanut2012/descargas.php

Egypt: https://dhsprogram.com/data/dataset/Egypt_Special_2015.cfm?flag=1

Ghana: https://apps.who.int/healthinfo/systems/surveydata/index.php/catalog/sage

India: https://dhsprogram.com/data/dataset/India_Standard-DHS_2015.cfm?flag=0

Indonesia: https://www.rand.org/labor/FLS/IFLS/access.html

Kyrgyzstan: https://dhsprogram.com/data/dataset/Kyrgyz-Republic_Standard-DHS_2012.cfm?flag=1

Lesotho: https://dhsprogram.com/data/dataset/Lesotho_Standard-DHS_2014.cfm?flag=1

Mexico: http://www.ennvih-mxfls.org/english/ennhiv-3.html

Namibia: https://dhsprogram.com/data/dataset/Namibia_Standard-DHS_2013.cfm?flag=0

Peru: https://dhsprogram.com/data/dataset/Peru_Continuous-DHS_2012.cfm?flag=1

Russia: https://apps.who.int/healthinfo/systems/surveydata/index.php/catalog/sage

Ukraine: https://dhsprogram.com/data/dataset/Ukraine_Standard-DHS_2007.cfm?flag=1

For the remaining countries, please contact Paul Martin at pmartin@hsph.harvard.edu

* For the member countries of the Caribbean Public Health Agency (CARPHA) - Belize, Grenada, Guyana and Saint Vincent and the Grenadines - data were shared through a Data Use Agreement signed with the Executive Director of CARPHA. The Senior Technical Officer for NCDs (Dr.Glennis Andall-Brereton) should be contacted in addition to Paul Martin, if necessary.
