# Supplementary material for: Association between country preparedness indicators and quality clinical care for cardiovascular disease risk factors in 44 lower- and middle-income countries: A multicountry analysis of survey data
Source: PLoS Med. 2020 Nov 10;17(11):e1003268. doi: 10.1371/journal.pmed.1003268 (PMC7654799; doi:10.1371/journal.pmed.1003268)
Supplement: S2 Text — (DOCX) [file pmed.1003268.s004.docx]

# **S2 Text. Data source and extraction method for facility readiness data**

National-level summary data were extracted from the World Health Organization (WHO) Service Availability Readiness Assessments (SARA - <https://www.who.int/healthinfo/systems/sara_introduction/en/>) and the Demographic and Health Survey (DHS) Service Provision Assessments (SPA - <https://dhsprogram.com/What-We-Do/Survey-Types/SPA.cfm>) for Burkina Faso, Kenya, Nepal, United Republic of Tanzania, Togo, and Uganda. However, of the six total SPA and SARA reports, data were only complete for availability of metformin and of insulin.

Surveys asked similar questions in different ways. These were extracted under one descriptor for each country, as follows: in Table 1, “percent of facilities with insulin” was asked as (translated from original language to English),

Burkina Faso 2014 SARA report: “percentage of availability of tracer elements for the diagnosis and/or treatment of diabetes among the establishments which offer the service: insulin injection”

Kenya 2013 SARA report: “percentage of facilities that have non-communicable disease tracer products observed in stock and valid: insulin injection”

Nepal 2015 SPA report: “among facilities offering services for diabetes, the percentages having indicated essential medicines observed at the service site on the day of the survey: injectable insulin”

Togo SARA 2012 report: “percentage of health facilities with tracer elements for diagnosis and / or management of diabetes among institutions offering this service: insulin injection”

Uganda 2014 SARA report: “percentage of facilities with insulin injection”

Tanzania 2013 SARA report: “among health facilities offering diabetes diagnosis and/or management services, the percentage with insulin injectable”

The indicator “percent of facilities with metformin” was asked as (translated from original language to English):

Burkina Faso 2014 SARA report: “percentage of availability of tracer elements for the diagnosis and/or treatment of diabetes among the establishments which offer the service: metformin”

Kenya 2013 SARA report: “percentage of facilities that have non-communicable disease tracer products observed in stock and valid: metformin cap/tab”

Nepal 2015 SPA report: “among facilities offering services for diabetes, the percentages having indicated essential medicines observed at the service site on the day of the survey: metformin”

Togo SARA 2012 report: “percentage of health facilities with tracer elements for diagnosis and / or management of diabetes among institutions offering this service: metformin”

Uganda 2014 SARA report: “percentage of facilities with metformin tablet”

Tanzania 2013 SARA report: “among health facilities offering diabetes diagnosis and/or management services, the percentage with metformin cap/tab”

The indicator “percent of facilities with glibenclamide” was asked as (translated from original language to English):

Burkina Faso 2014 SARA report: “percentage of availability of tracer elements for the diagnosis and/or treatment of diabetes among the establishments which offer the service: glibenclamide”

Nepal 2015 SPA report: “among facilities offering services for diabetes, the percentages having indicated essential medicines observed at the service site on the day of the survey: glibenclamide”

Togo SARA 2012 report: “percentage of health facilities with tracer elements for diagnosis and / or management of diabetes among institutions offering this service: glibenclamide”

Uganda 2014 SARA report: “percentage of facilities with glibenclamide tablet”

Tanzania 2013 SARA report: “among health facilities offering diabetes diagnosis and/or management services, the percentage with glibenclamide cap/tab”

The indicator “percent of facilities offering diabetes diagnostic and management services” was asked as (translated from original language to English):

Burkina Faso 2014 SARA report: “percentage of health facilities offering services for the diagnosis and / or treatment of diabetes”

Tanzania 2013 SARA report: “percentage of health facilities [offering] diabetes and/or management services”

Nepal 2015 SPA report: “among all facilities, the percentages offering services for diabetes: providers in the facility diagnose, prescribe treatment for, or manage patients with diabetes”

Togo SARA 2012 report: “percentage of facilities which offered services for the diagnosis and management of diabetes at the time of survey”

The indicator “among facilities offering diabetes diagnosis and management services, percent with at least one trained staff in diabetes diagnostic and management” was asked as (translated from original language to English):

- Burkina Faso 2014 SARA report: “percentage of availability of tracer elements for the diagnosis and/or treatment of diabetes among the establishments which offer the service: staff trained in diabetes diagnosis and treatment”
- Nepal 2015 SPA report: “among facilities offering services for diabetes, the percentages having at least one interviewed provider of diabetes services reported receiving in-service training in diabetes services during the 24 months preceding the survey. The training must have involved structured sessions; it does not include individual instruction that a provider might have received during routine supervision; observed to be available at the service site on the day of the survey”
- Togo SARA 2012 report: “percentage of health facilities with tracer elements for diagnosis and / or management of diabetes among institutions offering this service: staff trained in the diagnosis and treatment of diabetes during the two years preceding the survey”
- Tanzania 2013 SARA report: “among health facilities offering diabetes diagnosis and/or management services, the percentage with at least one trained staff diabetes diagnosis and treatment”

The indicator “among facilities offering diabetes diagnosis and management services, percent with guidelines for diagnosis and management” was asked as (translated from original language to English):

- Burkina Faso 2014 SARA report: “percentage of availability of tracer elements for the diagnosis and/or treatment of diabetes among the establishments which offer the service: guidelines for diagnosis and management of diabetes”
- Nepal 2015 SPA report: “among facilities offering services for diabetes, the percentages having guidelines for the diagnosis and management of diabetes observed to be available at the service site on the day of the survey”
- Togo SARA 2012 report: “percentage of health facilities with tracer elements for diagnosis and / or management of diabetes among institutions offering this service: diabetes diagnosis and treatment recommendations”
- Tanzania 2013 SARA report: “among health facilities offering diabetes diagnosis and/or management services, the percentage with guidelines available for diabetes diagnosis and treatment”

The indicator “among facilities offering diabetes diagnosis and management services, percent with blood pressure apparatus” was asked as (translated from original language to English):

- Burkina Faso 2014 SARA report: “percentage of availability of tracer elements for the diagnosis and/or treatment of diabetes among the establishments which offer the service: blood pressure apparatus”
- Nepal 2015 SPA report: “among facilities offering services for diabetes, the percentages having functioning digital blood pressure machine or manual sphygmomanometer with stethoscope observed to be available at the service site on the day of the survey”
- Togo SARA 2012 report: “percentage of health facilities with tracer elements for diagnosis and / or management of diabetes among institutions offering this service: blood pressure apparatus”
- Tanzania 2013 SARA report: “among health facilities offering diabetes diagnosis and/or management services, the percentage with blood pressure apparatus”

The indicator “among facilities offering diabetes diagnosis and management services, percent with blood glucose measurement capacity” was asked as (translated from original language to English):

- Burkina Faso 2014 SARA report: “percentage of availability of tracer elements for the diagnosis and/or treatment of diabetes among the establishments which offer the service: blood glucose”
- Nepal 2015 SPA report: “among facilities offering services for diabetes, the percentage having indicated diagnostic capacity: facility had a functioning glucometer and unexpired glucose test strips in the facility observed at the service site on the day of the survey”
- Togo SARA 2012 report: “percentage of health facilities with tracer elements for diagnosis and / or management of diabetes among institutions offering this service: blood glucose”
- Tanzania 2013 SARA report: “among health facilities offering diabetes diagnosis and/or management services, the percentage with ability to conduct test onsite and presence of glucometer and glucometer test strips”

The indicator “among facilities offering diabetes diagnosis and management services, percent with adult weighing scale” was asked as (translated from original language to English):

- Burkina Faso 2014 SARA report: “percentage of availability of tracer elements for the diagnosis and/or treatment of diabetes among the establishments which offer the service: weighing scale for adults”
- Nepal 2015 SPA report: “among facilities offering services for diabetes, the percentage having indicated equipment: adult weighing scale observed to be available at the service site on the day of the survey”
- Togo SARA 2012 report: “percentage of health facilities with tracer elements for diagnosis and / or management of diabetes among institutions offering this service: weighing scale for adults”
- Tanzania 2013 SARA report: “among health facilities offering diabetes diagnosis and/or management services, the percentage with adult scale”
