# Supplementary material for: Association between country preparedness indicators and quality clinical care for cardiovascular disease risk factors in 44 lower- and middle-income countries: A multicountry analysis of survey data
Source: PLoS Med. 2020 Nov 10;17(11):e1003268. doi: 10.1371/journal.pmed.1003268 (PMC7654799; doi:10.1371/journal.pmed.1003268)
Supplement: S3 Text — (DOCX) [file pmed.1003268.s005.docx]

# **S3 Text. Data source and extraction method for NCD readiness indicators**

NCD readiness indicators were collected for all countries where these reports were available within two years of the data collection for derivation of the cascades of care. Data were either extracted from the 2011 or 2014 WHO NCD Country Profile reports, whichever was closest to the year of conduct of the study from which cascades were derived. Countries where there were no reports available two years either side of the cascade data collection (n=5), were set to missing. There were slight variations in the way that questions were asked between years, as described in detail below.

Of the indicators presented in Table 1, “has an operational NCD unit/branch or department within the Ministry of Health, or equivalent” was asked as such in the 2014 report, and as “has a unit/branch/department in Ministry of Health with responsibility for NCDs” in the 2011 report.

The indicator, “has an operational policy, strategy or action plan to reduce physical inactivity and/or promote physical activity” was asked as such in the 2014 report, and as “has an integrated or topic-specific policy/programme/action plan which is currently operational for physical inactivity” in the 2011 report. The same wording was used for tobacco (reported as “burden of tobacco” in the 2014 report, alcohol (reported as “harmful use of alcohol” in the 2014 report), and diets (reported as “unhealthy diets and/or promote healthy diets” in the 2014 report and “unhealthy diet/overweight/obesity” in the 2011 report).

The indicator, “funding available for NCD surveillance, monitoring and evaluation OR for NCD treatment and control OR for NCD prevention and health promotion,” was derived as the aggregate of three different indicators, asked in 2011, which were “there is funding available for: NCD treatment and control”, “there is funding available for: NCD prevention and health promotion” and “there is funding available for: NCD surveillance, monitoring and evaluation”. None of these were asked in the 2014 report.

The indicators “has evidence-based national guidelines/protocols/standards for the management of major NCDs through a primary care approach,” “has an operational multisectoral national policy, strategy or action plan that integrates several NCDs and shared risk factors,” and “NCD surveillance and monitoring system in place to enable reporting against the nine global NCD targets” were only asked in the 2014 report, whereas the indicators “has an integrated or topic-specific policy, programme or action plan which is currently operational for cardiovascular diseases,” “has an integrated or topic-specific policy, programme or action plan which is currently operational for diabetes” were only asked in the 2011 report.
