# Supplementary material for: Association between country preparedness indicators and quality clinical care for cardiovascular disease risk factors in 44 lower- and middle-income countries: A multicountry analysis of survey data
Source: PLoS Med. 2020 Nov 10;17(11):e1003268. doi: 10.1371/journal.pmed.1003268 (PMC7654799; doi:10.1371/journal.pmed.1003268)
Supplement: S4 Text — (DOCX) [file pmed.1003268.s006.docx]

# **S4 Text. Data source and extraction for indicators of *country development*; indicators of *health service finance and equity*; and general indicators of *health service performance or readiness***

All indicators were extracted by March 31^st^ 2019 and were extracted for the year for which data for constructing the care cascade were available. Apart from Human Development Index, downloads were from the World Bank World Development Indicators repository (data.worldbank.org). All data are updated annually. Original data sources, where different, are detailed below.

*Country development indicators*

Gross Domestic Product (GDP) per capita data is compiled by the World Bank based upon their national accounts data and those of the Organisation for Economic Cooperation and Development (OECD; https://data.oecd.org).

Human Development Index (HDI) is compiled by the United Nations Development Programme. It incorporates life expectancy at birth, mean years of schooling for adults (or expected years of schooling for children), and gross national income per capita (<http://hdr.undp.org/en/content/human-development-index-hdi> )

Indicators of *health service finance and equity*

Current Health Expenditure (%GDP), Domestic private health expenditure (% of current health expenditure), and Out-of-pocket expenditure (% of current health expenditure) are collected by WHO and compiled in the Global Health Expenditure database ([apps.who.int/nha/database](http://apps.who.int/nha/database)).

*Health system performance or readiness indicators*

Neonatal mortality rate (per 1,000 live births) is estimated by the UN Inter-agency Group for Child Mortality Estimation (UN IGME – including UNICEF, WHO, World Bank, and UN DESA Population Division) and can be found at [childmortality.org](http://www.childmortality.org)

Hospital beds (per 1,000 people) data are aggregated by World bank from WHO sources and supplemented by individual country data.

Physicians (per 1,000 people), and nurses and midwives (per 1,000 people) are aggregated by World Bank based upon WHO’s Global Health Workforce Statistics (https://www.who.int/hrh/statistics/en/), OECD data (https://data.oecd.org), and supplemented by individual country data
