# Supplementary material for: Association between country preparedness indicators and quality clinical care for cardiovascular disease risk factors in 44 lower- and middle-income countries: A multicountry analysis of survey data
Source: PLoS Med. 2020 Nov 10;17(11):e1003268. doi: 10.1371/journal.pmed.1003268 (PMC7654799; doi:10.1371/journal.pmed.1003268)
Supplement: S5 Text — (DOCX) [file pmed.1003268.s007.docx]

**S5 Text. Model specification and Stata code for mixed-effects logistic regression**

To determine the association of indicators with each of the six clinical care outcomes, we used maximum likelihood estimation of mixed-effects logistic regression models and robust variance estimation. The sample for each regression was all individuals with diabetes or, for the three clinical care outcomes specific to hypertension, all individuals with hypertension. All models were adjusted for country, specified as a random effect, and individual-level predictors, specified as fixed effects (age, sex, and education).

More specifically, the models fitted can be conceptualized as follows.

$\mathrm{logit}\left\{ E\left( Y_{ij}|X_{i},V_{i} \right) \right\}=\beta_{0}+ \beta_{1}X_{i}+\beta_{2}{Age}_{ij}+\beta_{3}{Sex}_{ij}+\beta_{4}{Education}_{ij}+V_{i}$

where

*Y_ij_* (0 or 1) is the binary outcome variable for the *j*th participant (*j* = 1, 2, …, *n_i_*) in the *i*th country (*i* = 1, 2, …, *K*)

*X_i_* is the indicator of interest and is fixed within a country, but can vary between countries (e.g., GDP per capita, hospital beds per 1,000 people, etc.)

*β_0_* is a constant representing the baseline log-odds

*V_i_* is the random effect specific to the *i*th country

All models were estimated using Stata v. 14.2 (StataCorp, College Station, Texas, US). Generic code, for example, for the indicator “GDP” as a predictor of the outcome “hypertension (HTN) diagnosis,” was as follows:

meglm HTNdiagnosis zGDP zAge sex i.education [pweight=weight] if hypertension==1 | | country:, fam(bernoulli) link(logit) vce(robust)

When vce(robust) is specified, robust variances are clustered at the highest level in the multilevel model, which, in our models, was country.
