# Supplementary material for: Association between country preparedness indicators and quality clinical care for cardiovascular disease risk factors in 44 lower- and middle-income countries: A multicountry analysis of survey data
Source: PLoS Med. 2020 Nov 10;17(11):e1003268. doi: 10.1371/journal.pmed.1003268 (PMC7654799; doi:10.1371/journal.pmed.1003268)
Supplement: Attachment — Submitted filename: Response to reviewers comments 08-04-20.docx [file pmed.1003268.s008.docx]

COLLEGE OF MEDICAL

AND DENTAL SCIENCES

Institute of Applied Health Research

Dr Justine Davies MD, MRCP, MBChB, BMSc, BSc, DipABRSM

Professor of Global Health

Institute for Applied Research

Birmingham University

Birmingham

UK

B15 2TT

+44 (0)7384584673

@drjackoids

<https://www.birmingham.ac.uk/staff/profiles/applied-health/justine-davies.aspx>

4^th^ April 2020

Re: Revision of PMEDICINE-D-19-04192.

Dear Editors and reviewers,

Please find below the response to editors’ and reviewers’ comments

We look forward to hearing from you in due course

Kind regards

Justine Davies, on behalf of all authors

Responses to comments from the editors:

General comment: Please include line numbers in the revised version of your manuscript.

*This has been done*

General comment: There are a number of typographical errors in your manuscript; please proofread carefully prior to resubmission.

*The manuscript has been proof read and these have been corrected*

General comment: Please cite reference numbers in square brackets, leaving a space before the reference bracket, and removing spaces between reference numbers where more than one reference is cited e.g. “... delivery of quality clinical care to patients [1,2].”

*This has been done*

Ethics Statement: Please provide evidence of the determination of “not human subjects research” by the institutional review board of the Harvard T.H. Chan School of Public Health on 9 May 2018.

*The official letter from the institutional review board is attached.*

Data Availability Statement: PLOS Medicine requires that the de-identified data underlying the specific results in a published article be made available, without restrictions on access, in a public repository or as Supporting Information at the time of article publication, provided it is legal and ethical to do so. Please see the policy at <http://journals.plos.org/plosmedicine/s/data-availability> and FAQs at

<http://journals.plos.org/plosmedicine/s/data-availability#loc-faqs-for-data-policy>

An appropriate contact (URL or email address) will be required for data access. Please note that this cannot be a study author.

*We have added the following Data Availability Statement to the title page:*

*“This study includes individual-level data from 44 countries. Some of this data is publicly available and other data are available by request to the country. We have arranged specific data use agreements for surveys that are not publicly accessible and we are unable to share these data given the terms of our agreements. S1 Text provides a complete list of country-contacts through which those data which are not publicly available can be requested and accessed.”*

Please revise your title according to PLOS Medicine's style, placing the study design in the subtitle (ie, after a colon). We suggest “Association between country preparedness indicators and quality clinical care for cardiovascular disease risk factors in 44 lower and middle income countries: A multi-country analysis of survey data” or similar.

*This has been done*

Abstract Background:

*The abstract has been thoroughly revised in line with the comments, below. However, this has resulted in an increase in the word count of the abstract. We can reduce the number of words by substantially summarising the findings; however, would prefer to leave the granularity in this section if possible.*

Please expand upon the context of why the study is important. The final sentence should clearly state the study question.

*This has been done*

Please combine the Methods and Results components of your Abstract into one subsection titled ‘Methods and Findings’

Please include the study design, brief demographic details of the population studied (e.g. age, sex) and further details of the study setting (e.g. patient data collected from where? Hospitals/community care? How many low v middle income countries?), number of individuals included and main outcome measures.

Please specify the dates between which data was used in the analysis (months and years)

*This has been done*

Please revise ‘ quality-clinical-care’ into separate words

*This has been done*

In the last sentence of the Abstract Methods and Findings section, please describe the main limitation(s) of the study's methodology.

*This has been done*

Please begin your Abstract Conclusions with "In this study, we observed ..." or similar. Please address the study implications, emphasizing what is new without overstating your conclusions.

*This has been done*

At this stage, we ask that you include a short, non-technical Author Summary of your research to make findings accessible to a wide audience that includes both scientists and non-scientists. The Author Summary should immediately follow the Abstract in your revised manuscript. This text is subject to editorial change and should use non-identical language distinct from the scientific abstract. Please see our author guidelines for more information: <https://journals.plos.org/plosmedicine/s/revising-your-manuscript#loc-author-summary>

*This has been done*

Please ensure to include a final bullet point under ‘What do these findings mean?’ to describe the main limitations(s) of the study.

*This has been added, as below:*

***“Author Summary***

*Why was the study done?*

- *Diseases, for example, high blood pressure and diabetes are becoming increasingly common in low and middle income countries (LMICs, or so-called, “developing” countries).*
- *Treatment for these conditions is simple and cheap. However, without treatment, people with them are at high risk of suffering adverse consequences, such as heart attacks and strokes.*
- *It is important therefore to be able to measure whether patients who need treatment are getting it. Currently, LMICs’ progress towards being able to treat patients with hypertension and diabetes is measured using proxies, for example, whether policies, guidelines, funding, structures, or human resources are in place.*

*What did the researchers find?*

- *We measured whether 187,552 people with hypertension living in 43 LMICs and 40,795 people with diabetes living in 28 LMICs had their high blood pressure or diabetes treated well, i.e. they had these conditions diagnosed, treated, or controlled.*
- *We found that most proxy measures were not reflective of whether patients had their condition treated well.*

*What do these findings mean?*

- *To judge countries’ progress towards ability to treat hypertension and diabetes requires directly assessing whether people with these diseases are getting the treatment that they need.*
- *The main limitation of the study was that a one-time measurement of blood pressure or blood glucose was used to define whether participants had diabetes or high blood pressure. To make a concrete clinical diagnosis requires more detailed investigation****.****”*

Introduction

Please expand upon the need for and potential importance of your study.

*We have inserted the following into the introduction to show the important context of our study*

*“Cardiovascular diseases are one of the most common causes of death and disability in lower and middle income countries (LMICs). Reducing premature mortality from these conditions is a key aim of the Sustainable Development Goals (SDG 3.4).[1] To ensure that this goal is met requires identifying and adequately treating people who have conditions like diabetes and hypertension. Monitoring country progress towards meeting this goal is essential to ensuring commensurate investment in health systems to manage these conditions.”*

Indicate whether your study is novel and how you determined that.

In the sentence beginning ‘But achievement of quality clinical care…’ please add ‘to our knowledge’, and begin sentence with ‘However…’

*The authors on this study know the relevant literature well, in addition, we did a scoping review of the literature to assess whether any study had previously been done to associate clinical care quality outcomes for cardiovascular risk factors with proxy measures used to monitor health services.*

*We have added mention of that to the sentence below, as underlined. That sentence also implies that the study is novel (“not previously been associated with…”). If the editors would prefer us to explicitly state the study is novel, we are happy to change the language:*

*“However, author knowledge and review of the literature showed that achievement of quality clinical care to manage prevalent disease across multiple countries has not previously been associated with indicators commonly used to assess performance of the health services or NCD readiness indicators.”*

Please revise sentence beginning ‘Also, to assess whether or not these …’ to ‘We also aimed to assess…’ or similar

*This has been done.*

Methods

Did your study have a prospective protocol or analysis plan? Please state this (either way) early in the Methods section. If a prospective analysis plan was used in designing the study, please include the relevant prospectively written document with your revised manuscript as a Supporting Information file to be published alongside your study, and cite it in the Methods section. If no such document exists, please make sure that the Methods section transparently describes when analyses were planned, when/why any data-driven changes to analyses took place, and what those changes were.

*We have adjusted the methods to make the analysis plan clearer (see response to Reviewer 2, point Ma2.4). We have also included the following text in the methods:*

*“**None of the analyses presented in this paper were prespecified. The decisions to exclude the indicators, (1) operational multisectoral national policy for NCDs and shared risk factors and (2) NCD surveillance and monitoring system in place to enable reporting against the nine global NCD targets, and to evaluate the correlation of country wealth and health expenditure were made during data analysis. All other analyses were planned.”*

Please ensure that the study is reported according to the STROBE guideline, and include the completed STROBE checklist as Supporting Information. Please add the following statement, or similar, to the Methods: "This study is reported as per the Strengthening the Reporting of Observational Studies in Epidemiology (STROBE) guideline (S1 Checklist)."

The STROBE guideline can be found here: <http://www.equator-network.org/reporting-guidelines/strobe/>

When completing the checklist, please use section and paragraph numbers, rather than page numbers.

Please remove italicisation from your Methods section.

*This has been done*

Results

Please include a separate table of demographic information of the population studied (including total number of individuals included, age, sex, education, etc) as Table 1, including numerators and denominators for percentages where possible.

*We have put this as table 3, with tables 1 and 2 describing the characteristics of other data sources and indicators used in this analysis.*

Please clarify whether 43 or 44 LMICs were included (you state 44 in the title, but 43 were included for hypertension)

*43 of these 44 countries had data on hypertension. The 44^th^ (Fiji) had data on diabetes but not on hypertension.*

Please refer to individual components of your supplementary materials e.g. Table S1 rather than page numbers within the supplementary file

*This has been done*

Please provide units for GDP per capita

*This has been done*

Please clarify what is meant by ‘of current’ with regard to private health / out of pocket expenditure

*We have added the following text to the first instance where that term is used:*

*“Note, for out of pocket expenditure, “of current” refers to how the metric is reported by the source. For each country, data were extracted for the year in which the cascade survey was done, thus, “current” refers to that year.”*

Please clarify whether reference to ref 11 is intended in sentence beginning “Corresponding figures for diabetes were…”

*Apologies – this has been removed*

Please provide some numerical data from Tables 3 & 4 in the main test of your Results section, when outlining main findings.

*This has been done*

Please make clear what numbers presented in the main text of your Results refer to e.g. “strongly associated with achieving hypertension control (2·11[1·10 - 4·05])” should include RR

*These corrections have been made throughout*

Tables 2 & 3 - please ensure that it is made clear what values represent e.g. %, n, RR (95% CI) etc.

*This has been done*

Discussion

Please present and organize the Discussion as follows: a short, clear summary of the article's findings; what the study adds to existing research and where and why the results may differ from previous research; strengths and limitations of the study; implications and next steps for research, clinical practice, and/or public policy; one-paragraph conclusion.

Please add ‘to our knowledge’ to the sentence beginning “Very little, if any, previous research…”

*We have extensively revised the entire discussion to structure as advised. We have not included the text here, as the entire discussion has been changed.*

Please provide evidence for the post-hoc analysis (in accordance with the PLOS data policy) within the Results section.

*This has been done*

*“Post-hoc analyses done to explore relationships between country wealth and health expenditure showed GDP was significantly associated with OOP expenditure (Pearson correlation coefficient, -0.34, p=0.02), but not significantly associated with lower private health care expenditure (Pearson correlation coefficient, -0.26, p=0.09).”*

References

Please ensure that all references are appropriately and consistently formatted and capitalised e.g. ref 10 Jama should be JAMA, ref 24 should be PLoS Med, ref 34 Chatham House, etc.

*This has been done*

Responses to comments from the reviewers:

Reviewer #1:

1.1This well-written paper is concerned with the relevance of preparedness indicators to some clinical care outcomes. Specific comments are given below.

The model that was used in this paper is somewhat unclear, perhaps the authors could write it explicitly in an appendix, including whether the outcomes were allowed to correlate and any potential multicollinearity issues. For example, the lack of significance for some associations could possibly be attributed to collinearity issues.

Assuming a random effects logistic regression was used it would be useful to add the estimation method, since this does matter outside the linear mixed model framework.

If a Bayesian approach was used please add the priors for the variance components.

*"**To determine the association of indicators with each of the six clinical care outcomes (condition 1, diagnosed, 2, treated, or 3, controlled for both diabetes and hypertension), we used separate mixed-effects Poisson regression models with a binary outcome (indicating whether or not the individual achieved the clinical care outcome) and robust standard errors. Model parameters were estimated using maximum likelihood. The sample for each regression was all individuals with diabetes or, for the three clinical care outcomes specific to hypertension, all individuals with hypertension. All models were adjusted for country, specified as a random effect, and individual-level predictors, specified as fixed effects (age, sex, and education). These individual-level predictors have previously been shown to be predictive of progress through the cascades of care."*

Also please add the details of the rescaling process.

*We have re-written this sentence as follows to clarify how the sample weights were rescaled:*

*"**Regression analyses took into account sample weights, which were rescaled by dividing an individual's sample weight by the sum of weights of respondents in the estimation sample such that all sample weights within the sample summed to 1 and thus all countries contributed equally to the overall estimates."*

There is a large numbers of comparisons/tests conducted in this paper, how was multiplicity accounted for?

*This is certainly a relevant comment. The issue of multiplicity is quite common in applied health systems research such as this study, and is concerning because if there are truly no effects for some of the indicators tested, by using multiple tests we may have identified statistically significant effects that are not in fact real. Given that the indicators for preparedness evaluated in this study were selected a priori, and specifically chosen because they were hypothesized to be associated with quality clinical care outcomes, we did not think it would be appropriate or necessary to account for multiplicity. Moreover, given that we found few statistically significant effects, and our conclusion is that these indicators are poor proxies for quality clinical care received by patients, we are not especially concerned with making Type 1 errors.*

In relation to this comment, please discuss the model and variable selection procedure.

*We have re-written the statistical analysis section to clarify what model was used (a mixed-effects Poisson regression with MLE). To ensure comparability of findings across indicators, the variables included in the models were the same for all indicators: (1) fixed effects for individual-level factors (sex, age, and education) previously shown to be highly predictive of progress through the cascades of care and (2) random effects for country.*

1.2 Clinical care is generally hard to measure so it would be useful to discuss the relevance of the selected outcomes, the associated uncertainty any potential measurement error and their influence on the statistical analysis.

*We agree that, in general, clinical care is hard to measure. However, for diabetes and hypertension, there are agreed-upon thresholds for treatment and control which can readily be used to indicate whether quality clinical care outcomes (i.e. the patient has been treated correctly according to guidelines) have been achieved.*

*The surveys which were used to create the care cascades in this analysis were done using methodologies and measurement devices that have been chosen and recommended by international agencies (for example, WHO or USAID) based upon their field measurement reliability; they are well accepted for assessing prevalence in epidemiology studies.*

*That said, and as reviewer 4 also alludes to, making a clinical diagnosis of hypertension or diabetes requires more detailed investigation. For example, one needs at least two elevated blood pressure readings on separate, consecutive occasions to meet a diagnosis of hypertension, and definitive diagnosis of diabetes requires oral glucose tolerance testing.*

*In large scale epidemiology surveys, such as the WHO STEPS surveys - which we mainly used - these methods are not feasible and the survey’s results are accepted in the literature as reliable estimates (see R2R references: 1-6). Nevertheless, we recognise that our population estimates of diabetes or hypertension prevalence may have been either inflated (in the case of hypertension where lack of respondent familiarity with having blood pressure measured can elevate blood pressure) or under-estimated (in the case of diabetes, where an oral glucose tolerance test may have picked up more people with diabetes). These could both lead to errors in estimates of the proportions of people who we defined as having the disease or being controlled.*

*We have added the following to the limitations section:*

*“The survey data which we used to complete the cascades of care are epidemiology studies which rely on field data rather than formal clinical diagnoses of hypertension or diabetes. Formal clinical diagnoses require more detailed investigation. For example, at least two elevated blood pressure recordings on separate, consecutive occasions are required to meet a diagnosis of hypertension, and definitive diagnosis of diabetes requires oral glucose tolerance testing. In large scale epidemiology surveys, these methods are not feasible, and although the survey’s results are accepted in the literature as reliable estimates, we recognise that these may be over or under-estimates.[11-13, 16, 31].*

*However, our aim in this paper is not to assess access to quality clinical care (our previous studies have reported these findings)(1, 3), but rather to explore associations of health-service-relevant factors with markers of quality clinical care. The errors in estimating cascade steps are likely to be the same or very similar across all countries included in the study and minimised in cross country comparisons”.*

1.3 The message of the paper is clear. However, it would be useful to discuss all the reasons that the  preparedness indicators are collected for and whether dropping them would affect any other current process.

*Our response also addresses some of the points raised by reviewer 4.*

*The preparedness indicators that are the focus of this paper (the “NCD readiness indicators”) are collected by WHO to assess country progress towards achieving global NCD targets. CVDRF-related indicators are only one part of this data collection. However, diabetes and cardiovascular diseases form two out of four of the major NCDs assessed by this report. These preparedness indicators are described in the main text. Details on other preparedness indicators have been added to the appendix.*

*We agree with the need to comment upon whether dropping any indicators from global data collection would affect other current processes and have added reflections on this to the discussion. In general, we are not arguing for preparedness indicators to be dropped, rather that they should not be the first consideration when assessing health system’s readiness to manage CVDRFs. As we state in the manuscript:*

*“It may be more efficient to use indicators that are reflective of patient care outcomes as the first step in a health service assessment, with health service markers used to define where barriers to care are in countries that don’t meet certain quality clinical care targets – perhaps defined as those that are achievable in other settings with well-functioning health services.”*

*We have further clarified that we are referring to health service assessment for CVDRF in our analysis and that the indicators that we used may have utility for assessing health service preparedness to deal with other conditions, although their utility for other conditions needs further exploration.*

*We have also added the following to the discussion:*

*“It should be noted that some of the indicators used in this study are also collected for other monitoring purposes, and although we have shown their lack of utility as a proxy for good clinical care outcomes for CVDRF, they may have utility for these other purposes. That utility should be tested in further studies”.*

Reviewer #2: Thank you for the opportunity to review this manuscript. I commend the authors for undertaking this ambitious analysis as it helps to address an important gap in the evidence base on the monitoring and evaluation of global health systems for NCD control. I have listed below several minor and major comments.

Minor comments:

Mi2.1.	Please clarify if the levels (%) of each outcome reported in Table 2 were also weighted in a similar way when pooling across countries for the regression models? If not, why not?

*Yes, all countries were weighted equally in summarizing country-level indicators in Table 3 – the same approach as the regression models. We have added this note to the footnote of Table 2:*

*“All countries contributed equally to these estimates.”*

Mi2.2.	In the results section, some of the figures in the text do not include units, and there is inconsistent labelling of estimates.

*This has been corrected.*

Mi2.3.	On page 13 paragraph 4, it seems that reference 11 is cited in the text, but it is unclear why.

*This was an error; the reference has been removed*

Mi2.4.	There are some issues with punctuation throughout.

*We have re-read the manuscript and corrected spelling and grammatical errors throughout.*

Mi2.5.	For figure 1, I would suggest using a coding scheme that is suitable when printing in grey scale to help better distinguish between categories.

*Thank-you, this has been noted. We also note that PLoS Medicine publish figures in colour and we would be happy to be advised by the production team regarding colour schemes for figures that also may be seen in grey scale.*

Major comments:

Ma2.1.	I agree strongly with many of the key statements in the discussion section, including that there is little evidence suggesting that indicators of service readiness are reflective of health system performance or success - which is the main motivation for this analysis, and that direct assessments of achievement of cascade steps for hypertension and diabetes management may be the most reliable ways of judging equitable care. On this second point, one publication where this has been done for hypertension is <https://doi.org/10.1186/s12939-016-0478-6>.

*Thank-you, we have added that reference to our discussion*

Ma2.2. However, to better substantiate such points, including several statements made in the manuscript's concluding paragraph, I suggest that much deeper engagement with the supposed relationships between indicators on various health system inputs and the cascade step outcomes is needed. For example, the introduction section could present information on why proxy indicators are often used in lieu of difficult to measure concepts, including patient-level measures, and how good proxy indicators are selected and justified, such as through the development and application of conceptual frameworks (e.g. the PHCPI conceptual framework: <https://improvingphc.org/phcpi-conceptual-framework>).

*We have added some text about this to the introduction, as follows:*

*“Many of these indicators are proxies for patient outcomes that are based on conceptual frameworks, such as the Primary Healthcare Performance Initiative’s Conceptual Framework of critical elements of a strong primary healthcare system. These are often based upon the Donabedian “structures” and “processes” needed to deliver care. Although these outcomes may be relatively easy to measure, the utility of some of them to reflect quality patient clinical care outcomes (the Donabedian “outcomes”) has been called into question, especially as many are based largely on expert opinion or feasibility of collection”*

Ma2.3. For this analysis, a conceptual framework that relates a range of health system input proxies to the gold standard indicators (i.e. cascade steps) could help to identify which are likely to be good candidates (i.e. theoretically, readiness indicators that are more proximal to the cascade steps should be better proxies), and could serve as the basis for test if these relationships are borne out empirically, which is essentially what the analysis presented in this manuscript attempts to do. A conceptual framework could also provide possible explanations for some of the observed associations readiness indicators and cascade steps. For example, a readiness indicator such as medicine availability may be strongly associated with treatment and control because it is proximal to the cascade step outcome, or weakly associated because there is lots of heterogeneity or effect modification - despite being proximal to the cascade step.

Similarly, a very distal indicator, such as GDP per capita or HDI, may still be strongly associated because, much like the cascade step, it may reflect the summative effects of all the various pathways through which a health system impacts risk factor control in individuals. But ultimately, because the way that health systems achieve better levels of risk factor management both for individuals and populations is multi-factorial, with many interlinked causal pathways operating across several levels, this means how success is achieved will vary considerably by context, and an understanding of how each jurisdiction makes such achievements is needed. Therefore, a conceptual model that maps readiness indicators to cascade steps could help to identify any gaps that may be hindering better risk factor management as targets for policy intervention, which is a point that is brought up in the current introduction text. While I do not suggest that the manuscript be modified to include such in-depth analysis, I do think that addressing

such points introduction and discussion could result in more nuanced, sound and meaningful interpretations of the findings.

*Thank-you for these really interesting comments. Indeed, we started this research with exactly the concept that this reviewer alludes to and we have made the methods clearer in this regard.*

*We created a high-level working framework based upon the author’s knowledge of health systems (in particular, informed by Atun’s framework (7) – see below), available data, and diabetes and cardiovascular disease care. We then collated variables collected by numerous agencies and mapped to this framework those that authors considered most relevant. To avoid overfitting, we limited the number of variables at each stage in our framework after discussion amongst authors.*

*Our framework and its variables were hierarchical. We started with the country context in which the health system sits, hypothesising that a strong health system to enable care for complex chronic conditions, like diabetes and hypertension, would be more likely in developed countries or those with a higher GDP (hence variables of country development, GDP and human development index. At the next level were high-level health system factors focused on financial spending on health (these are captured in the following three variables: domestic government expenditure on health [as % GDP], or % private or % out of pocket expenditure on health [as % current]). Equity is also reflected in % private or % out of pocket spending on health. In this hierarchical framework, markers of NCD preparedness sit with high-level health system factors as they reflect countries’ commitment to facilitate quality clinical outcomes for diabetes and hypertension.*

*More proximate to the patient are markers of health services readiness or preparedness, here hospital beds (per 1,000 people), nurses and midwives (per 1,000 people), or physicians per 1,000 people; and neonatal mortality rate (per 1,000 live births). Closer to patient care are facility readiness indicators to provide specific care. Finally, we included individual participant characteristics.*

*We have adjusted the methods to make this clearer, as below:*

*“We conceptualised a high-level working framework based upon the author’s knowledge of health systems (in particular, informed by Atun’s 2013 framework for analysis of Turkey’s health system) [19], available rigorously collected data, and CVDRF care. We then collated numerous variables collected by health and development agencies and mapped to this framework those that authors agreed were most relevant. Where there were multiple variables within these data sources of potential relevance to the study question, those selected were chosen (on discussion between JD, SK, LJ, LH, PG, and JMG) based on relevance to the question, avoidance of duplication, and availability within two years of the data used to construct the care-cascade. Given that care delivery for CVDRF has not changed rapidly in many LMICs, it was decided that the two year threshold allowed reasonable temporal comparability between data sources.*

*Our framework was hierarchical. We started with the country context in which the health system sits, hypothesising that a strong health system to enable care for complex chronic conditions, like CVDRF, would be more likely in countries with a higher Human Development Index (HDI) or those with a higher GDP (markers of country development). In this hierarchical framework, markers of NCD readiness sit with high-level factors as they reflect countries’ commitment to facilitate quality clinical outcomes for CVDRF. At the next level were high-level factors focussed on financial spending on health and equity (indicators of health service finance and equity). More proximate to the patient are indicators of health service performance (whether a health service is delivering care) or readiness (whether it is able to deliver care). Health facility readiness indicators reflect facility readiness to provide specific care for CVDRF. Lastly are the patient-proximate indicators of individual-level patient characteristics.”*

*We have also adjusted the way the results are reported throughout the manuscript to be consistent with this structure.*

Ma2.4.	In the introduction, the authors draw attention to the high level of resources needed to collect national service readiness data, but it should also be acknowledged that collecting nationally representative data on blood pressure, blood glucose levels, current medication status and health histories of individuals is also resource intensive, and may be even more so than the effort needed to collect service readiness data. In a sense, this has been used to partly justify the value of the SARA data collection, but also underscores the need to identify reliable and cost-effective proxy indicators, if possible.

*Again, thank you for this comment, we agree that collection of nationally representative data on clinical outcomes can be resource intensive. However, we would argue that it is more efficient use of time to collect these data as a first step than to base assumptions of quality care on proxies – for example SARA surveys. If clinical care outcomes are found wanting, CVDRF modules can be easily added to surveys, e.g: SARA, to ascertain where gaps are. At the moment, given SARA or NCD readiness markers are not reflective of quality clinical care for CVDRF, they are, unfortunately, not cost-effective for assessing these conditions.*

Ma2.5.	 In the methods, could you please clarify if year dummies were included in models as, in some countries, patient-level data were collected over several years, and across countries these could help to control for potential regional or global time-dependent effects (e.g. changes to treatment guidelines, such as from mono- to combination therapy) as the included survey data span 2005 to 2016. And if not, why not. Similarly, given that there are expected (unobserved) differences across countries that would likely confound the associations of interest, my preference would be to use country fixed effects, rather than a random effect for country in the multi-level specification. So some justification for the use of a random effect is needed.

*We did not include year dummies in the models. Only one survey was included per country, and only one country collected data in certain years, and so we cannot tease apart differences between countries vs. differences between years. However, we do not see this as a major limitation as we suspect that whilst there will be differences in the absolute values of the indicators and clinical care outcomes over time, the association between the indicators and clinical care outcomes is likely to be constant and it was this association that was of interest in this study. We chose to model the country effects as random effects because we were interested in estimating fixed effects of country-level indicators (listed in Table 1, e.g. GDP per capita, current health expenditure, physicians per 1000 population , etc.) and if country fixed effects were used instead, the country-level indicator would be absorbed into the country fixed effect.*

Ma2.6.	A key issue in the discussion section is that it does not address the important role played by private health care providers, including retail pharmacies, as key sources of care for both hypertension and diabetes in LMICs. An important limitation of the supply-side data sources used is that, while both the SPA and SARA surveys do include private health facilities, they do not capture retail pharmacies and other possible sources of private care outside of formal health facilities (i.e. hospitals and clinics).

*Associating facility level markers and outcomes was an exploratory analysis in this study as we only had information from six countries and only on health facility readiness to provider diabetes care. However, we have noted this important limitation in the limitations section of our manuscript, as follows:*

*“SARA and SPA surveys do not capture information from retail pharmacies or other places of care delivery outside of the formal sector; these providers are often used by patients with CVDRF, but unfortunately our data on medications availability did not cover them”.*

Ma2.7. Given the proximity of indicators for medicine availability to the cascade outcomes in the causal relationship, one would expect these to be among the most strongly associated indicators with diabetes treatment and control, but were only found to be 'approaching significance'. [As an important aside, such interpretations should be avoided throughout the manuscript in preference for the reporting p-values so that readers may assess the strength of the associations themselves.]

*We have removed any text suggesting that associations approached significance.*

Two possible explanations for this unexpected observations follow from my comments above: 1) because the supply-side data does not capture medicine availability from other important sources of private care as described above,

*Please see above*

or 2) the analytical approach used 'over aggregates' medicine availability data when producing national-level indicators from the micro-data, which are then regressed against the individual-level outcomes data. Such aggregation eliminates important cross-cluster heterogeneity of indicators within countries, thus reducing the ability to detect strong associations. Palafox et. al (<https://doi.org/10.1016/j.ssmph.2019.100376>) note the limitations of measuring access to care/health system performance (or even indicators of quality care) in this way, and demonstrate an alternative method that merges contemporaneous patient-and facility-level data (such as DHS and SPA data) to produce more realistic and people-centred measures.

*We agree that this is a limitation of this (exploratory) part of our analysis and have added the following to the limitations section:*

*“Additionally, our aggregation of individual patient clinical outcomes and SARA or SPA data at a national level may mask some positive associations between these variables which may be seen if these were available to be analysed at a local level,* *but data were not available for that level of study”.*

Ma2.8. Perhaps following a similar approach (e.g. regressing aggregate measures of patient- and facility-level outcomes at cluster level in countries with DHS and SPA data) would produce stronger evidence of an association.

*We agree that the approach that the reviewer suggests for associating facility readiness for CVDRF with clinical care (cascade) outcomes would be interesting. However, the main reason for our analysis was to explore whether national level NCD readiness indicators were associated with quality clinical care outcomes. Associations between facility readiness and outcomes was an exploratory analysis.*

*Also, further analyses associating SPA/SARA level data with clinical quality care outcomes would be limited by data availability, as mentioned, above, and quality. Where local linkage is possible between DHS and SPA/SARA, the DHS data are often present but for hypertension only (for example, Ukraine or Azerbaijan), or self-reported (for example, the Maldives).*

*See above addition to the limitations*

Reviewer #3: This was an interesting and comprehensive manuscript that could potentially inform countries preparedness on cardiovascular diseases risk factors.

I enjoyed reading your manuscript. However, as a discretionary comment I would like the authors to add a line about why some countries with availability of DHS data on hypertension  and diabetes were excluded from the study for example Ukraine, Azerbaijan, Armenia, Maldives etc were not included in this study.

*Thank-you for your positive comments on our manuscript. We have included all data sources that were available to us and where data was of high enough quality to use. Both Ukraine and Azerbaijan, for example, were included. As far as we are aware, the DHS survey in Maldives (2009) collected self-reported data on respondents’ experiences with blood pressure (e.g. if they had their blood pressure measured, if they had been diagnosed with high blood pressure, treatment, etc.) but did not actually measure blood pressure. In order to be eligible for this study, a country had to have a measurement of blood pressure or a measurement of a glucose biomarker. Data from Armenia were not cleaned by the time the data were locked for analysis.*

Reviewer #4: Association between country preparedness indicators and quality clinical care for cardiovascular disease risk factors in 44 lower and middle income countries

Foremost, I would like to appreciate the team for conceptualising and investing a heavy amount of time in generating evidence for association between country preparedness indicators and quality of clinical care for cardiovascular disease outcomes in 44 lower and middle income countries. This study is worth the effort and would give us a lot of insights if it is conducted rigorously.

*Thank you for this comment. We have conducted the study as rigorously as possible and have added comments to your specific points below to further clarify.*

Unfortunately, I find several limitations that makes it difficult for me to agree with the current conclusion drawn by the authors.

4.1 Foremost, the authors do not give us a detailed account of how the indicators are collected, when they are collected and whether they are appropriately collected? It is possible that poorly collected data is being used to make strong inferences/conclusions? If this is the case, one would question the associations? Is it a true association or cofounded outcome due to poorly collected data for the various indicators?

*All of the indicators used in this analysis are from well-respected sources e.g: UN, WHO, World Bank, and are as rigorously collected or estimated as current methodologies allow. They have been used extensively in other analyses. Importantly, we have avoided using modelled data in this analysis.*

*In response of the need for a more detailed account of how the indicators are collected, multiple indicators, collected by multiple different agencies are used in this analysis and to give a full account of the methodologies used by those agencies would markedly add to the word count of the paper and appendices. We have added text to the appendices to show the original source of each variable and a link to where these are held and where more information can be found, as follows:*

***“Text S3. Data source and extraction for indicators of country development; indicators of health service finance and equity; and general indicators of health service performance or readiness***

*All indicators were extracted by March 31^st^ 2019 and were extracted for the year for which data for constructing the care cascade were available. Apart from Human Development Index, downloads were from the World Bank World Development Indicators repository (data.worldbank.org). All data are updated annually. Original data sources, where different, are detailed below.*

*Country development indicators*

*Gross Domestic Product (GDP) per capita data is compiled by the World Bank based upon their national accounts data and those of the Organisation for Economic Cooperation and Development (OECD; https://data.oecd.org).*

*Human Development Index (HDI) is compiled by the United Nations Development Programme. It incorporates life expectancy at birth, mean years of schooling for adults (or expected years of schooling for children), and gross national income per capita (*[*http://hdr.undp.org/en/content/human-development-index-hdi*](http://hdr.undp.org/en/content/human-development-index-hdi) *)*

*Indicators of health service finance and equity*

*Current Health Expenditure (%GDP), Domestic private health expenditure (% of current health expenditure), and Out-of-pocket expenditure (% of current health expenditure) are collected by WHO and compiled in the Global Health Expenditure database (*[*apps.who.int/nha/database*](http://apps.who.int/nha/database)*).*

*Health system performance or readiness indicators*

*Neonatal mortality rate (per 1,000 live births) is estimated by the UN Inter-agency Group for Child Mortality Estimation (UN IGME – including UNICEF, WHO, World Bank, and UN DESA Population Division) and can be found at* [*childmortality.org*](http://www.childmortality.org)

*Hospital beds (per 1,000 people) data are aggregated by World bank from WHO sources and supplemented by individual country data.*

*Physicians (per 1,000 people), and nurses and midwives (per 1,000 people) are aggregated by World Bank based upon WHO’s Global Health Workforce Statistics (https://www.who.int/hrh/statistics/en/), OECD data (https://data.oecd.org), and supplemented by individual country data”*

*We have likewise already summarised the data collection for the cascades that were constructed for this analysis. This is in the study methods. Given that the cascades themselves have been published elsewhere, we have referred the reader to those initial papers (in PLoS medicine and The Lancet) for more detailed methodologies.*

4.2 Another important pitfall has to do with the cascade data. The authors use cross sectional survey data collected at community level as proxy for measuring cascade outcomes - "diagnosis", "treatment" and "control". I don't think this is correct. We know very well that cross sectional survey at the community level for estimating for example hypertension are screening activities and give a glimpse of the possible disease burden but not necessarily diagnostic. Thus, this data normally skews the estimates.

*Please see our response to reviewer #1, point 1.1 for a full response. We re-iterate here that whilst we agree with these comments, and have added some text to the limitations to address this, the epidemiological data that we have used in this study are considered to be robust and the best methodology we have at present time for estimating disease burden and access to care for individual patients with diseases in LMICs. Related responses can also be found below under response to comment 4.3*

4.3 I would imagine the most accurate outcomes should be drawn at the health facility level where diagnosis, care treatment etc. is provided. Unless we have cascaded programs combining community and health facilities, the current study might be interpreted as misleading, first of all, given the diverse sources of data but also the point at which the outcomes is measured.

*More accurate data are found in health facilities for people living in High Income Countries, like the UK, where a large proportion of the population regularly visit healthcare facilities and practitioners are incentivised to regularly measure outcomes and collect accurate data. Unfortunately, the situation in many LMICs, where this study is focused, does not allow for this.*

*First, in most LMICs, health facility data are paper-based and not widely available to enable comparisons across (or even within) countries.*

*Second, combining community studies with health facility studies would not solve the issue in question as in most LMICs a large section of the population doesn’t access healthcare. Even if they did, one would still need to define the population prevalence of disease in order to detect those who have the condition, but who have not accessed care. Most epidemiology studies that determine population prevalence do so by using the methods that we have outlined (indeed, 44 countries in our study have used these methods). Very few epidemiology studies do “facility quality” testing for diabetes and hypertension in the field (as this would entail visiting participants on two separate occasions for measurement of blood pressure or administration of a time-consuming oral glucose tolerance test). Those studies that have used this rigorous methodology– e.g: The PURE study - have only done this in a limited number of countries.*

*Third, combining community studies with healthcare facility data can also be troublesome given that in many LMICs, community members don’t show fidelity to one particular service provider and may travel for large distances to access care. This is certainly what we have experienced in our work in Burkina Faso, Sierra Leone, and South Africa, for example.*

*In sum, we start our cascade with the step “diagnosed,” which necessitates asking people if they have received a diagnosis for their condition. This is relevant to understand whether people with the condition in the community have been given a diagnosis which would facilitate them accessing treatment. If one starts at the health facility, one misses this first crucial step as people who are attending a health facility for that condition would generally have been given a diagnosis for it. The alternative is to use health facility data to assess the proportion of people attending the health facility and with an abnormal reading for blood pressure or blood glucose who have had that abnormality documented as a clinical condition. This is certainly an interesting question, but one which we feel should be the subject of future research.*

4.4 I would also like to bring to the attention of the authors about the proximal and dismal nature of the various indicators. For example, having an operational policy, strategy or action plan to … I wonder how operational is measured? I can report "yes" or "no" to these indicators and yet in reality only a handful of facilities/communities in a country have these policies operational. This is particularly true at least for Uganda that I am familiar with. Making some of these policies operational is still a challenge as much as they might be reported as operational. So such indicators would only be helpful if we track their operationalization slightly a little further.

*We agree wholeheartedly with this statement, which is one of the initial reasons for wishing to do this study. Countries are currently being evaluated on their progress towards achieving global targets for NCDs (within the SDGs and the UN High Level Commission on NCDs). The NCD readiness indicators which we use in our study and which this reviewer alludes to are high level (or distal) indicators. Nevertheless they are regularly collected by WHO and published in order to show country “[capability] to respond to the epidemic of noncommunicable diseases.” We demonstrate in our analysis that these (and other indicators of service delivery or readiness) are not sufficient to judge progress towards these targets. Progress should be judged by attainment of good quality clinical care. It would be tremendously interesting to do as this reviewer suggests and track operationalization of these indicators further, but that is the subject of future study and one which we hope will gain traction after publication of our study.*

4.5 On the other hand, we have indicators such as number of physicians, nurses, or hospital beds per 1000 people etc. These are more likely to be collected accurately. Moreover, they are proximal indicators, so linking them to quality of care takes a shorter path than the former. No wonder the authors found "number of physicians was associated with being treated and controlled (1.12 [1.06 - 1.17] and 1.10 [1.01 - 1.20], respectively).  In summary the outcome - quality clinical-care has a fairly complicated causal/non causal pathway that should be well thought through to generate inferences.

*Thank-you, these comments align with those from reviewer 2 (Ma2.3). We have added more on the high-level conceptual framework used in our study to the methods. This includes mention of the proximity of these measures to patient healthcare delivery, relative to the more distal measures of country development or NCD readiness. However, these more proximate measures were interestingly not associated with better clinical care outcomes for diabetes and we cannot draw the conclusion that their proximity to patient care is therefore the reason for the positive association with hypertension cascade outcomes.*

*We have added some text to the discussion around this (underlined below)*

*“It is possible that these measures are better associated with clinical care outcomes as they are more proximate to patient care delivery than the NCD readiness indicators, however, that associations were not consistent suggests that further work needs to be done on the utility of these indicators before accepting them as truly reflective of health service functioning”.*

4.6. Another challenge that I find with the current study is the multiple sources of data used to manipulate the associations. The authors need to clearly describe the data bases and also possible generate a schema on how the different data bases were managed for analysis.

*As per comment 4.1, we have added text on the data sources to the Appendix.*

*Table 1 outlines data categories, variables, source, and number of countries with available data.*

4.7. Authors infer to the field of maternal and new-born health, and use this as a basis for the current concept.  However, the papers they quote are methodically incomparable, (1, 23 and 24), and thus it is difficult to draw similar conclusions.

*Although the methods are different, the concepts are similar – both studies ask whether proxy measures of care reflect quality clinical care outcomes, both conclude that the associations are not straightforward (and imply that the Donabedian framework of Structures, Processes, and Outcomes, is not linear or causal).*

*In reference 27, Leslie and colleagues use granular facility level data from 8 countries to associate infrastructure and quality for maternal, child health, and family planning services. Our paper asks the related question of do “higher-level” health service variables positively associate with quality clinical care for CVDRF. Our conclusions are the same as in reference 27. They are no less relevant, given that proxy variables – whether collected at national, system, or service level - are widely taken to reflect progress to providing quality outcomes.*

*Ours is the first study to look at this issue for CVDRF, and in combination with others’ work in fields related to MDG targets, the evidence strongly points to the need to find new ways of measuring quality.*

*As we have discussed in our response to reviewer 2 (Ma2.8), granular data are not yet available to allow multi country associations with clinical care quality at an individual facility level.*

4.8. Therefore, with the current data sources as analysed, it is difficult to accurately predict country preparedness and quality clinical care outcomes for cardiovascular disease risk factors (CVDRF). More work is needed to assess the pathways. For the different indicators and how they are interlinked to each other.

*We agree with this statement, however, our aim was not to predict country preparedness for quality clinical care outcomes for CVDRF. To address that question would require complex causal modelling approaches, and even with their use, we may find that there is no magic recipe that allows one to predict preparedness or determine factors which are the lynchpins for providing quality care.*

4.9. I also need to state that maternal and neonatal data is currently one of the well collected data in most of these low income countries. Operationalization of guidelines is almost unquestionable and the data is fairly if not accurately tracked right through the healthcare delivery levels. It is still a struggle for NCD data though there is some effort.

*We also agree with this statement, as we have reflected upon above in response to your comment 4.7. Interestingly, despite the operationalization of guidelines being unquestionable in the field of maternal and neonatal health, Leslie et al (our reference 27) still found a disconnect between proxy markers and provision of good quality clinical care.*

Finally, I don't seem to appreciate how the authors particularly addressed the "process" strand of the Donabedian's framework. This is particularly important in measuring quality outcomes.  If we say we have guidelines, it is not enough?  How many of these guidelines have trickled down?

*As we have now better explained in our methods – see response to reviewer 2’s point (Ma2.1) – our framework was not based upon Donabedian’s, but we have used that in the text to illustrate the broad concepts thought necessary for delivery of quality clinical care (“outputs”). Our conceptual framework was based upon that of Atun et al (Lancet 2013), and involved a degree of pragmatism in the selection of data.*

*Processes are captured in the NCD readiness variables on guidelines, and the reviewer is correct in that these may not have trickled down to patient care level. Nevertheless, we have shown that when they are available, they are not affecting improvement in clinical care quality. Processes are also captured in the SARA/SPA data where facilities report on the availability of guidelines for diabetes care and whether staff have been trained (which could, one could argue, reflect trickle down of the guidelines). Unfortunately, neither of these variables was associated with quality clinical care outcomes.*

*References used in these responses:*

1.	Geldsetzer P, Manne-Goehler J, Marcus M-E, Ebert C, Zhumadilov Z, Wesseh CS, et al. The state of hypertension care in 44 low-income and middle-income countries: a cross-sectional study of nationally representative individual-level data from 1&#xb7;1 million adults. The Lancet.

2.	Manne-Goehler J, Atun R, Stokes A, Goehler A, Houinato D, Houehanou C, et al. Diabetes diagnosis and care in sub-Saharan Africa: pooled analysis of individual data from 12 countries. The Lancet Diabetes & Endocrinology. 2016;4(11):903-12.

3.	Manne-Goehler J, Geldsetzer P, Agoudavi K, Andall-Brereton G, Aryal KK, Bicaba BW, et al. Health system performance for people with diabetes in 28 low- and middle-income countries: A cross-sectional study of nationally representative surveys. PLOS Medicine. 2019;16(3):e1002751.

4.	Collaborators GBDCoD. Global, regional, and national age-sex-specific mortality for 282 causes of death in 195 countries and territories, 1980-2017: a systematic analysis for the Global Burden of Disease Study 2017. Lancet. 2018;392(10159):1736-88.

5.	Worldwide trends in diabetes since 1980: a pooled analysis of 751 population-based studies with 4·4 million participants. The Lancet. 2016;387(10027):1513-30.

6.	Basu S, Wagner RG, Sewpaul R, Reddy P, Davies J. Implications of scaling up cardiovascular disease treatment in South Africa: a microsimulation and cost-effectiveness analysis. The Lancet Global health. 2019;7(2):e270-e80.

7.	Atun R, Aydin S, Chakraborty S, Sumer S, Aran M, Gurol I, et al. Universal health coverage in Turkey: enhancement of equity. Lancet. 2013;382(9886):65-99.
