# Supplementary material for: Association between country preparedness indicators and quality clinical care for cardiovascular disease risk factors in 44 lower- and middle-income countries: A multicountry analysis of survey data
Source: PLoS Med. 2020 Nov 10;17(11):e1003268. doi: 10.1371/journal.pmed.1003268 (PMC7654799; doi:10.1371/journal.pmed.1003268)
Supplement: Attachment — Submitted filename: Response to reviewers comments 08-06-20.docx [file pmed.1003268.s009.docx]

COLLEGE OF MEDICAL

AND DENTAL SCIENCES

Institute of Applied Health Research

Dr Justine Davies MD, MRCP, MBChB, BMSc, BSc, DipABRSM

Professor of Global Health

Institute for Applied Research

Birmingham University

Birmingham

UK

B15 2TT

+44 (0)7384584673

@drjackoids

<https://www.birmingham.ac.uk/staff/profiles/applied-health/justine-davies.aspx>

8^th^ June 2020

Re: Revision of PMEDICINE-D-19-04192.

Dear Editors and reviewers,

Please find below the response to the remaining reviewers’ comments

We look forward to hearing from you in due course

Kind regards

Justine Davies, on behalf of all authors

Reviewer #1:

The authors have revised their paper and this is a substantially improved manuscript.

However, one major issue remains.

This relates to the selection of a Poisson regression model with a binary dependent variable.

If I'm guessing correctly (given that the authors still did not explicitly added their model) the logarithm of the probability of achieving the clinical care outcome is linked to the fixed and random effects of the model.

This creates an unnecessary constrain as the log-probability is inherently negative, likely yielding biased estimates.

One would think that the canonical version, assuming a Bernoulli distribution with a logit (or probit or cloglog) link ought to be used instead, avoiding the potential biases of a Poisson/binary model.

If this guess is correct it seems reasonable to re-run all the analyses using the canonical model.

This may or may not significantly affect the results, but it will remove some biases from what is an already challenging model.

Also, please add the details of the estimation method, including for the variance components (was that done using restricted maximum likelihood?), and write the model explicitly in the supplement, to avoid any further confusion

*Thank you for this suggestion. In order to address the reviewer’s comment, we have re-run all of the analyses using a Bernoulli distribution with a logit link. As hypothesized by the reviewer, this did not substantially affect the results. We have retained this suggested model for simplicity. We have also included the model explicitly in the supplement.*

Reviewer #3: Thank you for taking time to address the reviewers' comments, the manuscript is much stronger now and my comments have been addressed satisfactorily.

*Thank- you for this comment*

Reviewer #4: The authors have reasonably addressed my comments. They may still need to check for typos e.g line 152 should be rely and not reply.

*Thank-you for this comment, we have thoroughly read through the manuscript and corrected a couple of minor typos detected*
